# Supplementary material for: Content-rich biological network constructed by mining PubMed abstracts
Source: BMC Bioinformatics. 2004 Oct 8;5:147. doi: 10.1186/1471-2105-5-147 (PMC528731; doi:10.1186/1471-2105-5-147)
Supplement: Additional File 2 — The original results of the above study (non-essential files are deleted to keep the file size under the limit set by BMC bioinformatics). [file 1471-2105-5-147-S2.bz2 › chilibotAdditionalFile2/dip10/662ID9346309E11055/html/CCR1_CCL15.html]

 


 **CCR1** and **CCL15** 
  
Found 16 abstracts in PubMed, retrieved 10.  
 

 What does Google say? 
 PDF only 
| .edu only 

---

**Interactive relationship** (e.g. stimulation, inhibition, etc)

**Neutral relationship**- Leukotactin 1  [ **CCL15** ]   **CCL15**  induced chemotaxis signaling through  **CCR1**  in HOS cells.  Ref: 11943214 FEBS Lett, 2002
- Leukotactin 1  [ **CCL15** ]  Lkn 1  [ **CCL15** ]   **CCL15**  is a recently cloned CC chemokine that binds to the  **CCR1**  and CCR3.  Ref: 11943214 FEBS Lett, 2002
- Differential effects of leukotactin 1  [ **CCL15** ]  and macrophage inflammatory protein 1 alpha on neutrophils mediated by  **CCR1** .  Ref: 10202040 J Immunol, 1999
- We conclude that  **CCR1**  is a receptor mediating responses to both MIP 1 alpha and Lkn 1  [ **CCL15** ]  on neutrophils and produces different biological responses depending on the ligand bound.  Ref: 10202040 J Immunol, 1999
- Leukotactin 1  [ **CCL15** ]  Lkn 1  [ **CCL15** ]  is a human CC chemokine that binds to both CC chemokine receptor 1  [ **CCR1** ]   **CCR1**  and CCR3.  Ref: 11832479 J Biol Chem, 2002
- CC chemokine receptor 1  [ **CCR1** ]   **CCR1**  that is known to be a receptor for Lkn 1  [ **CCL15** ]  and CKbeta8 1 in neutrophils, monocytes, and lymphocytes, was also present on the surface of CD34 cells from CB.  Ref: 12803479 Mol Cells, 2003
- In contrast,  **CCL15**  is characterized using molecular forms deduced from the mRNA cDNA and shown to activate cells via  **CCR1**  and CCR3, also dependent on the amino terminal length.  Ref: 11527984 J Leukoc Biol, 2001
- The human CC chemokine leukotactin 1  [ **CCL15** ]  Lkn 1  [ **CCL15** ]  is both a strong chemoattractant for neutrophils, monocytes, and lymphocytes and a potent agonist for  **CCR1**  and CCR3.  Ref: 10202040 J Immunol, 1999
- Human CC chemokine 2 HCC 2  [ **CCL15** ]  is a novel member of the chemokine peptide family that induces chemotaxis of monocytes, T lymphocytes and eosinophils via activation of the CCR 1  [ **CCR1** ]  and CCR  [ **CCR1** ]  3 receptors.  Ref: 10604595 J Pept Res, 1999

**Non-interactive relationship** (e.g. studied together, co-existance, homology, etc.)

- To understand the intracellular events following Lkn 1  [ **CCL15** ]  binding to  **CCR1** , we investigated the activities of signaling molecules in response to Lkn 1  [ **CCL15** ]  in human osteogenic sarcoma cells expressing  **CCR1** .  Ref: 12759139 Life Sci, 2003
- MIP 1alpha and MIP 5  [ **CCL15** ]  were found to be the most effective chemoattractants,  **CCR1**  was the main receptor expressed and modulated during LPS treatment, and MIP 2, RANTES, IP 10 and MCP 1 were the chemokines modulated during DC maturation.  Ref: 10360972 Int Immunol, 1999
- ... neutrophils from  **CCR1**  mice failed to respond to either MIP 1 alpha or Lkn 1  [ **CCL15** ] .  Ref: 10202040 J Immunol, 1999
- To understand the mechanism of Lkn 1  [ **CCL15** ]  induced chemotaxis signaling, we examined the chemotactic activities of human osteogenic sarcoma cells expressing  **CCR1**  in response to Lkn 1  [ **CCL15** ]  using inhibitors of signaling molecules.  Ref: 11943214 FEBS Lett, 2002
